# Die Rolle der Medien bei Entstehung, Verlauf und Bewältigung von Essstörungen

**DOI:** 10.1007/s00103-020-03256-y

**Published:** 2020-12-04

**Authors:** Christina Peter, Hans-Bernd Brosius

**Affiliations:** Institut für Kommunikationswissenschaft, Ludwig-Maximilians-Universität München, Oettingenstr. 67, 80538 München, Deutschland

**Keywords:** Bulimie, Anorexie, Sozialer Vergleich, Kultivierung, Medienwirkung, Bulimia, Anorexia, Social comparison, Cultivation, Media effects

## Abstract

Essstörungen gehören in westlichen Gesellschaften zu den häufigsten psychosomatischen Erkrankungen. Die Medien werden seit geraumer Zeit dafür verantwortlich gemacht, einer der Auslöser von Essstörungen zu sein. Beispielsweise konnte in mehreren Studien gezeigt werden, dass Medien ein unrealistisches Schönheitsideal vermitteln und dieses gerade bei jungen Rezipientinnen eine Unzufriedenheit mit dem eigenen Körper bewirken kann.

Allerdings wurden 2 zentrale Aspekte bisher kaum betrachtet.

Zum einen fehlt es an Studien, in denen die Erkrankten selbst im Mittelpunkt stehen und die Rolle der Medien in der Entstehungs‑, Verlaufs- und Bewältigungsphase einer Essstörung betrachtet wird. Zum anderen gibt es kaum Untersuchungen dazu, wie und in welchem Umfang die Krankheit in den Medien thematisiert wird und wie solche Darstellungen auf die betroffene Gruppe wirken.

Auf Basis der bisherigen Forschung in diesem Bereich ist zu vermuten, dass die Wahrnehmung dessen, wie die eigene Krankheit in den Medien dargestellt wird, Konsequenzen für die Selbstwahrnehmung, das Wohlbefinden und das Handeln der erkrankten Personen hat. Der vorliegende Beitrag gibt einen Überblick über die komplexe Rolle von Medien im Rahmen einer Essstörung und zeigt vor allem Lücken in der kommunikationswissenschaftlichen Forschung zu diesem Thema auf.

## Abstract (en)

Eating disorders are among the most common psychosomatic illnesses in Western societies. For some time now, the media have been blamed as one of the potential triggers of problematic eating behavior. Several studies have shown that the media convey an unrealistic ideal of beauty and that this can cause dissatisfaction with one’s own body, especially among young recipients. However, two central aspects have not yet been thoroughly considered. First, there is a lack of research that focuses on people actually affected by an eating disorder and that considers the role of the media in the development, progression, and coping phase of an eating disorder. Second, there is hardly any research on how and to what extent the disease itself is addressed in the media and how such representations influence the affected group – in communication research, this is referred to as reciprocal effects. Based on previous research in this area, it can be assumed that the perception of how one’s own illness is portrayed in the media can have consequences for the self-perception, well-being, and actions of ill persons. This article provides an overview of the complex role of the media in the context of an eating disorder and, above all, highlights gaps in communication research on the topic.

## Einleitung

Essstörungen sind vor allem in westlichen Industrieländern verbreitete psychische Erkrankungen, die besonders (aber nicht ausschließlich) junge Erwachsene betreffen [1]. Die gängigste Form ist dabei die sogenannte Magersucht (Anorexia nervosa), gefolgt von Ess-Brech-Sucht (Bulimia nervosa) und der Binge-Eating-Störung (wiederkehrende Essanfälle). Auch wenn es unterschiedliche Zahlen zur Prävalenz der Erkrankungen gibt, so deutet besonders die vergleichsweise hohe Mortalitätsrate insbesondere von Magersucht (laut einer Metaanalyse von 1998 die höchste unter allen psychischen Erkrankungen, vgl. [2]) auf die Notwendigkeit hin, sich gesellschaftlich mit dem Thema auseinanderzusetzen. Allerdings gelten psychische Erkrankungen nach wie vor als Thema, über das nicht gerne öffentlich gesprochen wird; Betroffene fühlen sich stigmatisiert und fürchten um ihr gesellschaftliches Ansehen und als Grund hierfür wird oft die negative und stereotype Darstellung solcher Krankheiten in den Medien genannt [3, 4]. Erstaunlicherweise gibt es aber wenig empirische Befunde, wie und in welchem Umfang Essstörungen oder andere psychische Erkrankungen in den Medien dargestellt werden. Die Medien wurden bisher fast ausschließlich als möglicher Auslöser von Essstörungen in den Blick genommen: Die überwiegend experimentell ausgerichtete Forschung untersuchte dabei, wie sich idealisierte Körperdarstellungen in den Medien auf das Körperbild junger Rezipientinnen auswirken. Obwohl die Befundlage heterogen ist, überwiegen Studien, die negative Effekte zeigen (für einen Überblick siehe [5]).

Dabei ist die Rolle von Medien im Rahmen von psychischen Erkrankungen im Allgemeinen und bei Essstörungen im Speziellen deutlich komplexer. Zunächst können Medien tatsächlich, als ein Faktor in einem komplexen Geflecht von genetischen, sozialen und familiären Bedingungen, Mitauslöser für ein gestörtes Essverhalten sein. Hier ist also ihre Rolle in der Entstehungsphase der Erkrankung von Bedeutung. Darüber hinaus spielen sie im Verlauf und im Bewältigungsprozess eine Rolle – und zwar sowohl in positiver als auch in negativer Hinsicht: Vor allem Inhalte, die unrealistische Schönheitsideale transportieren, können sich negativ auf den Bewältigungsprozess auswirken – daneben können aber zum Beispiel digitale Angebote, Onlinecommunitys und Aufklärungsformate den Krankheitsverlauf auch positiv beeinflussen. Zuletzt haben gerade massenmediale Inhalte und vor allem auch Unterhaltungsformate einen Einfluss darauf, welches Bild sich die Gesellschaft von der Krankheit selbst und Personen mit Essstörungen macht. Alle 3 Bereiche erscheinen uns in einem Beitrag über den Zusammenhang zwischen Medien und Essstörungen als zentral, sodass wir auf diese Punkte im Folgenden näher eingehen möchten.

## Die Rolle von Medien in verschiedenen Phasen einer Essstörung

### Entstehungsphase: Medien(‑inhalte) als Ursache gestörten Essverhaltens

Ein Großteil der Forschung im Rahmen von Medien und Essstörungen beschäftigt sich mit Medien als potenziellen Auslösern bzw. Treibern für ein gestörtes Essverhalten. Ausgangspunkt dafür sind zunächst korrelative Beobachtungen: Studien zeigen, dass das in den Medien präsentierte Schönheitsideal in den letzten Jahrzehnten zunehmend schlanker wurde, obwohl der durchschnittliche Body-Mass-Index (BMI) von Frauen im gleichen Zeitraum anstieg, sodass Realität und Medienrealität immer weiter auseinanderklafften [6]. Parallel dazu gaben immer mehr Frauen an, unzufrieden mit ihrem eigenen Körper zu sein, und auch die Zahl an diagnostizierten Essstörungen stieg bis in die 1990er-Jahre stetig an. Durch diese Beobachtung drängte sich geradezu die Vermutung auf, dass die Medien für Unzufriedenheit und Essstörungen mitverantwortlich sein könnten. Eine solche Beobachtung allein sagt allerdings weder etwas über die Richtung eines möglichen Kausalzusammenhangs aus noch, dass dieser überhaupt existiert.

Entsprechend besteht die einschlägige Forschung zum Großteil aus Experimentalstudien sowie einigen darauf aufbauenden Metaanalysen (z. B. [7–9]). In den Untersuchungen werden meist junge Studentinnen in Stichprobenauswahl nach Verfügbarkeit („convenience samples“) mit idealisierten Körperdarstellungen (etwa Bilder von Werbemodels, vgl. [10, 11]) konfrontiert und danach zu ihrer Körperzufriedenheit und ähnlichen Konzepten, darunter oft auch die Neigung zu gestörtem Essverhalten, befragt. Als theoretische Grundlage fungieren dabei die „Theorie sozialer Vergleichsprozesse“ [12, 13] sowie der „Kultivierungsansatz“ [14–16]. Soziale Vergleiche werden im Alltag permanent, auch unbewusst vorgenommen: Wir vergleichen uns explizit oder implizit mit anderen Personen hinsichtlich verschiedener Merkmale wie Intelligenz, Aussehen, Reichtum, sozialer Position etc. Soziale Vergleiche fokussieren dabei auf situative Effekte: Beim Betrachten einer idealisierten Körperdarstellung in den Medien kommt es zum Aufwärtsvergleich (die andere Person wird als attraktiver wahrgenommen), der abhängig vom Individuum zu Frust oder Deprimiertheit über das eigene Aussehen/Körperbild führen kann oder aber dazu, dem Vorbild nachzueifern. Da diese präsentierten Schönheitsideale aber oft für den/die Normalbürger*in nicht oder nur schwer erreichbar sind (allein schon wegen eingesetzter Bildbearbeitungstechniken), kann Letzteres im schlimmsten Fall zu krankhaftem Verhalten führen [17].

Der Kultivierungsansatz nimmt längerfristige Effekte in den Blick [14–16]: Ursprünglich mit Bezug auf Gewaltdarstellungen im Fernsehen entwickelt, geht er im ersten Schritt davon aus, dass es eine Diskrepanz bzw. eine Verzerrung zwischen der tatsächlichen Realität und der Medienrealität gibt. Wie einleitend beschrieben, ist das im Bereich von Körperdarstellungen der Fall: Der durchschnittliche BMI einer in den Medien dargestellten Frau liegt deutlich unter dem durchschnittlichen BMI der westlichen Gesamtbevölkerung und diese Schere ging in den letzten Jahren auseinander [6]. Durch die ständige Konfrontation mit der Medienrealität fangen Rezipient*innen an, diese für die tatsächliche Realität zu halten und entsprechend das medial präsentierte Schönheitsideal zu verinnerlichen.

Die Befundlage zur Wirkung idealisierter Körperdarstellungen ist insgesamt heterogen, wobei Studien, die einen negativen Einfluss von idealisierten Körperdarstellungen auf die Zufriedenheit mit dem eigenen Körper zeigen konnten, mittlerweile überwiegen. Vor allem wurden bereits einige „boundary conditions“, also Rahmenbedingungen identifiziert, unter denen solche negativen Effekte verstärkt auftreten, wie etwa ein geringer Selbstwert, bestehende Unzufriedenheit mit dem eigenen Körper und ein sehr schlankes, internalisiertes Körperideal [8]. Diese Befunde legen nahe, dass die Medien in der Regel nicht als alleinige Ursache, sondern als Verstärker, Mediator oder Auslöser in dem Prozess einer potenziellen Erkrankung fungieren. Wie so oft liegt also keine einfache, einseitige *Stimulus-Response*-Wirkung (Medien verursachen Essstörungen) vor, sondern eher eine komplexe Wechselwirkung: Menschen mit bestimmten Prädispositionen und evtl. auch schon Neigung zu problematischem Essverhalten wenden sich verstärkt idealisierten Medieninhalten zu, die diese Problematik wiederum vergrößern^1^ [3].

Die Betrachtung von Medien(-inhalten) in der Entstehungsphase eines gestörten Essverhaltens dient vorrangig der Entwicklung präventiver Maßnahmen bzw. von Interventionsprogrammen. Man versucht herauszufinden, welche Personen besonders vulnerabel sind und welche Inhalte besonders problematisch sein könnten, um hier entsprechende Maßnahmen einzuleiten, etwa das Verbot oder die Kennzeichnung bestimmter Inhalte. So haben einige Länder bereits eine Kennzeichnungspflicht für bearbeitete Werbebilder eingeführt, etwa Israel und Frankreich. In Italien hat sich ein Großteil der Modelagenturen freiwillig dazu verpflichtet, keine Models mit einem untergewichtigen BMI zu beschäftigen. Da der BMI als Kriterium auf Kritik stieß, trat in Frankreich 2017 ein Gesetz in Kraft, demzufolge Models nur mit ärztlichem Attest beschäftigt werden können, um krankhaftes Untergewicht in der Branche zu verhindern. Auch wenn eine Evaluation dieser Maßnahmen noch aussteht, so konnten Experimentalstudien zeigen, dass die Präsentation normalgewichtiger Models im Vergleich zu untergewichtigen sich positiv (bzw. weniger negativ) auf das eigene Körpergefühl auswirkt [18, 19].

### Verlaufs- und Bewältigungsphase: Einfluss von Medieninhalten auf Betroffene

#### Erkrankte als Rezipient*innen

Wie im vorherigen Kapitel geschildert, wurde bisher hauptsächlich untersucht, ob Medien bei „gesunden“ Personen zur Ausbildung einer Essstörung beitragen können. Im Bereich der kommunikationswissenschaftlichen Forschung zu Medienwirkungen fehlen entsprechend Studien, die Effekte von medialen Inhalten auf bereits erkrankte Personen untersuchen. Bedenkt man allerdings, dass Essstörungen eine der höchsten Letalitätsraten unter allen psychischen Erkrankungen aufweisen [20], erscheint es dringlich, betroffene Personen selbst in den Fokus der Forschung zu stellen.

Ein Grund für diese Forschungslücke sind sicherlich auch forschungsökonomische Aspekte. Erkrankte sind eine spezielle, nicht ohne gewissen Aufwand zu erreichende Zielgruppe; dazu kommen ethische Abwägungen, inwiefern vulnerable Personen mit potenziell krankheitsverstärkenden Inhalten konfrontiert werden können (grundsätzlich gilt hier aber auch die Frage, ob und in welchem Umfang gesunde Personen mit Inhalten, von denen man ausgeht, dass sie gestörtes Essverhalten auslösen können, konfrontiert werden sollten). Auch im Rahmen von Befragungen ist es für Betroffene oft belastend, über das Thema zu sprechen. Trotzdem ist Forschung mit erkrankten Personen nicht zuletzt auch deshalb wichtig, um die Rolle von Medien bei der Entstehung eines essgestörten Verhaltens einschätzen zu können. Zwar können über Post-hoc-Befragungen und Tiefeninterviews keine Kausalzusammenhänge herstellt werden, sie helfen im Sinne der Methodentriangulation aber durchaus, experimentelle Befunde zu untermauern bzw. zu kontextualisieren, um die Rolle der Medien bzw. spezieller Inhalte als potenzielle Auslöser besser verstehen zu können.

Aber auch für den Verlauf bzw. die Bewältigung einer Essstörung können Medien eine zentrale Rolle spielen, und zwar sowohl in positiver wie auch in negativer Hinsicht. Hier geht es dann nicht mehr nur um die Wirkung von medialen Inhalten, sondern auch um deren gezielte Nutzung. Weitere methodische Zugänge könnten hier die Analyse von Nutzungsspuren im Internet (z. B. Aufruf bestimmter Informationsangebote) oder die Auswertung von Gesprächsprotokollen (z. B. aus Foren zum Thema) sein. Damit umgeht man das Problem, Betroffene direkt zu ihrer Krankheit befragen zu müssen, allerdings müssen natürlich auch hierbei ethische Gesichtspunkte, wie z. B. Datenschutz, berücksichtigt werden.

Eine der wenigen Arbeiten, die sich aus kommunikationswissenschaftlicher Perspektive umfassend mit der Rolle von Medien bei Essstörungen auseinandergesetzt hat, ist die Dissertation von Baumann aus dem Jahr 2009 [3]. In problemzentrierten Tiefeninterviews sprach die Autorin mit erkrankten Mädchen und jungen Frauen über die Rolle von Medien(-inhalten) in verschiedenen Phasen ihrer Erkrankung. Dabei stellte sich vor allem das Fernsehen als dominantes Medium noch vor Zeitschriften und Internet heraus. Medieninhalte zu den Themen Schönheit(-sideal), Ernährung und Essstörung selbst wurden von den Befragten häufig genannt. Insgesamt wurde die Rolle von Medien primär negativ bewertet und ihre Wirkung eher zu Beginn des Krankheitsverlaufs verortet, also als potenzieller Auslöser/Verstärker thematisiert. Die Betroffenen berichteten aber vor allem auch von einer relativ gezielten Zuwendung zu Inhalten, und zwar aus unterschiedlichen Gründen: „Medien werden sowohl zur Flucht vor der Krankheit genutzt als auch als Mittel zur Bewältigung sowie zur Aufrechterhaltung der Symptomatik gebraucht“ [3].

#### Therapeut*innen-Sicht

Neben der Selbsteinschätzung von betroffenen Personen kann die Einschätzung von Expert*innen eine wichtige zusätzliche Perspektive bieten, um die Relevanz von Medien(-inhalten) im Krankheitsverlauf zu beurteilen und sowohl konstruktives als auch destruktives Medienhandeln in diesem Zusammenhang besser zu verstehen. In ihrer Bachelorarbeit im Jahr 2017 hat Lochbihler^2^ Leitfadeninterviews mit Therapeut*innen geführt, die auf die Behandlung von Essstörungen spezialisiert waren. Diese stellten vor allem die Bedeutung des Internets im Allgemeinen bzw. sozialer Medien im Speziellen heraus, und zwar sowohl in Bezug auf konstruktives (also krankheitsbewältigendes) als auch destruktives (also krankheitsverstärkendes) Medienhandeln, wobei Letzteres aus Sicht der Therapeut*innen zu überwiegen scheint.

Ein angeführtes Beispiel sind sogenannte *Pro-Ana*-Foren, in denen Erkrankte (teils lebensgefährliche) Tipps zum Abnehmen und Verheimlichen der Erkrankung austauschen und sich gegenseitig beim Ausleben der Essstörung bzw. zu den dahintersteckenden Schönheitsidealen ermutigen [21]. Plattformbetreiber wie Instagram und Youtube versuchen diesen Trends durch die Sperrung entsprechender Hashtags entgegenzuwirken, allerdings berichten die Therapeut*innen von der Vernetzung Betroffener über private Whatsapp-Gruppen, die so gut wie nicht kontrollierbar sind.

Darüber hinaus werden zuletzt stark aufgekommene, „virale“ Trends in sozialen Netzwerken, die übertriebene Magerkeit propagandieren (z. B. [22]), als extrem problematisch erachtet. Bei potenziell gefährdenden Inhalten unterscheiden die Therapeut*innen zwischen essstörungsspezifischen und -unspezifischen Inhalten: Unter Erstere fallen Phänomene wie Pro-Ana, die direkt auf die Krankheit ausgerichtet sind; Zweitere umfassen vermeintlich neutralere Inhalte, die für gesunde Personen nicht im gleichen Maße problematisch sind (z. B. Informationen über Kalorien und Fitness, unterhaltende Formate wie „Germany’s Next Topmodel“) wie für Erkrankte, die solche Inhalte allerdings anders verarbeiten werden. Das Internet scheint aber durchaus auch konstruktiv genutzt zu werden, beispielsweise zur Selbstdiagnostik bzw. Suche nach therapeutischer Hilfe und Informationen über die eigene Krankheit.

Zusammenfassend zeigt sich aus der Einschätzung der Therapeut*innen eine eher aktive Mediennutzung mit dem Fokus auf Onlinemedien, die sich thematisch eng an der Krankheit orientiert und vor allem von den Motiven nach Orientierung und Austausch getrieben wird. Aber auch die ständige passive Konfrontation mit medialen Schönheitsidealen über Werbung und andere Formate wird für den Bewältigungsprozess als problematisch erachtet. Formate wie „Germany’s Next Topmodel“ werden von den Expert*innen kritisch gesehen, weil sie suggerieren, Schlanksein und Attraktivität wären die einzigen Faktoren für Erfolg und Anerkennung gerade bei Frauen. Übereinstimmend mit den Befunden von Baumann [3] sehen auch Expert*innen eine starke Zuwendung von Erkrankten zu Medieninhalten, die sich mit den Themen Aussehen und Gewicht beschäftigen. Solche Inhalte sind also, wie bereits angesprochen, nicht nur potenziell krankheitsauslösend – Personen mit entsprechenden Dispositionen wenden sich solchen Inhalten auch verstärkt zu.

## Die mediale Darstellung von Essstörungen und ihre Wirkung auf Erkrankte

Sowohl in den Tiefeninterviews mit Betroffenen [3] als auch in den Therapeut*innen-Interviews^3^ kristallisierte sich heraus, dass Personen mit gestörtem Essverhalten Medien auch häufig nutzen, um sich über die eigene Krankheit zu informieren bzw. Berichte darüber besonders aufmerksam verfolgen. In diesem Zusammenhang stellt sich die Frage, welche Rolle Essstörungen als Krankheitsbild eigentlich in den Medien spielen. Hier lohnt sich, neben dem Blick auf Gesundheitsinformationen im Internet, schon allein aufgrund der Reichweite die Betrachtung massenmedialer Inhalte. Hier gibt es für den deutschsprachigen Raum nur wenige und vor allem keine aktuellen Befunde; Baumann et al. [4] konnten in ihrer Inhaltsanalyse der Presselandschaft von 2000 zeigen, dass sich das Thema Essstörung in unterhaltenden Formaten wie Boulevard- und Frauenzeitschriften, aber auch durchaus oft in Qualitätszeitungen wiederfindet. Dabei wird Anorexie deutlich häufiger thematisiert als andere Formen (obwohl die Zahl der an Bulimie erkrankten Menschen für gewöhnlich deutlich höher liegt, [1]).

Zusätzlich wäre interessant, wie das Thema in anderen Mediengattungen dargestellt und verhandelt wird, etwa in unterhaltenden Formaten wie aktuellen Streamingserien oder Filmen, die von der entsprechenden Zielgruppe stark rezipiert werden. Gerade hier wäre auch das Potenzial für *Entertainment-Education*, also die Verknüpfung von Unterhaltung und bildenden Inhalten, besonders hoch [23, 24].

Neben dem rein quantitativen Umfang ist von Interesse, welches Bild in den Medien von Essstörungen allgemein und speziellen Unterformen (z. B. Magersucht oder Bulimie) gezeichnet wird. Auch wenn für diesen speziellen Bereich kaum Untersuchungen vorliegen, kann aufgrund verwandter Forschung im Bereich Gesundheitskommunikation vermutet werden, dass hier vor allem dramatische und emotionale Einzelfälle Eingang in die Berichterstattung finden [25]. Das liegt zum einen daran, dass Boulevardzeitungen/-zeitschriften vorrangig prominente oder besonders drastische Fälle aufgreifen und diese dramatisierend ausführen [4]; zum anderen zeigen aber auch Befunde der Fallbeispielforschung [26], dass Journalist*innen bei der Schilderung von sozialen Phänomenen/Problemen/Erkrankungen generell gerne auf Einzelfälle zurückgreifen und hierbei besonders dramatische oder emotionale Geschichten bevorzugen [27–29]. Auch Baumann und Kollegen [4] konnten in ihrer Inhaltsanalyse zeigen, dass Betroffene selbst einen Großteil der dargestellten Akteur*innen in der Berichterstattung ausmachen.

Hier stellt sich die Frage, wie solche Darstellungen dann wiederum auf von Essstörungen betroffene Personen wirken. Aufbauend auf dem „Modell reziproker Effekte für soziale Gruppen“ [30, 31] und unter Erweiterung durch das Phänomen der Wahrnehmungsverzerrung bei der Medienrezeption [32] sowie Überlegungen zu sozialen Vergleichsprozessen [13, 33] möchten wir hier einige empirisch noch zu verifizierende Vermutungen ableiten. Das Modell reziproker Effekte beschäftigt sich mit der Wirkung von Berichterstattung auf die dort dargestellten Personen selbst. Zunächst wurde das Modell in Bezug auf Einzelpersonen entwickelt [28], später aber auch auf soziale Gruppen wie etwa Migrant*innen oder Rechtsextreme übertragen [30]. Basierend auf den Annahmen des Modells sowie ausgehend von den oben dargestellten Befunden ist davon auszugehen, dass Betroffene die Berichterstattung intensiver als andere verfolgen. Die bisherige Forschung lässt vermuten, dass erkrankte Personen dabei die mediale Darstellung negativ verzerrt wahrnehmen, und zwar umso stärker, je mehr sie sich mit der Gruppe der Erkrankten insgesamt identifizieren (Hostile-Media-Phänomen; [34]). Gleichzeitig stellen die Betroffenen Vermutungen an, wie die Darstellung der Krankheit in den Medien auf andere wirkt – je negativer die Darstellung wahrgenommen wird, desto stärkere Effekte werden in der Regel auf andere Rezipient*innen unterstellt [35, 36]. Darauf basierend führen sie das Verhalten ihres Umfelds ihnen gegenüber auf die mediale Darstellung zurück und richten wiederum ihr eigenes Verhalten an dem wahrgenommenen Einfluss aus [37].

In der bisherigen Forschung zur wahrgenommenen Medienwirkung auf Dritte wurde vorwiegend die soziale Nähe zu anderen als Einflussvariable untersucht; bei der Untersuchung von psychischen Erkrankungen erscheint eine weitere Unterscheidung notwendig, und zwar zwischen „Eingeweihten“ (also Personen, die von der Erkrankung wissen) und „Nichteingeweihten“. Wird die Darstellung der Erkrankung in den Medien als sehr negativ wahrgenommen, könnte das die Redebereitschaft den Eingeweihten gegenüber erhöhen (um sich zu rechtfertigen), gleichzeitig aber die Bereitschaft senken, sich weiteren Personen zu offenbaren.

Eine Schwäche des Modells zu reziproken Medieneffekten ist, dass es durch den Fokus auf soziale Gruppen Effekte auf das Selbstbild des Individuums weitestgehend ausblendet, diese aber gerade bei psychischen Erkrankungen wie einem essgestörten Verhalten von enormer Bedeutung sind. Wood und Kolleg*innen [38] konnten schon früh zeigen, dass in den Medien porträtierte, an der gleichen Krankheit leidende Personen wichtige Vergleichsstandards für Betroffene darstellen (unter anderem weil direkter Kontakt mit anderen Erkrankten oft fehlt). Deshalb erscheint es sinnvoll, das Modell um den Forschungsstand zu sozialen Vergleichsprozessen im Rahmen der Mediennutzung zu erweitern [13]. Demnach gleichen erkrankte Personen die mediale Darstellung der Krankheit mit ihrer eigenen Situation ab und bewerten, ob es ihnen selbst besser, schlechter oder ähnlich geht. Dies kann – je nachdem, ob es zu Assimilations- oder Kontrasteffekten kommt – positive oder negative Folgen haben. Kontrasteffekte kommen zustande, wenn die Lage einer Vergleichsperson nicht erreichbar erscheint [39]: Aufwärtsvergleiche mit anderen Erkrankten, denen es besser geht, können dann etwa Frustration oder Deprimiertheit auslösen, wohingegen Abwärtsvergleiche mit schlechter gestellten Personen zur Erleichterung über die eigene Situation führen.^4^ Letzteres hat zwar dann einen unmittelbaren positiven Effekt auf das Selbstbild, könnte allerdings dazu führen, die Krankheit nicht hinreichend ernst zu nehmen (im Sinne von „so schlecht wie dem/der geht’s mir ja nicht!“, [13]). Darüber hinaus können Aufwärtsvergleiche mit anderen Betroffenen aber auch motivierend wirken, etwa wenn gezeigt wird, wie die Essstörung erfolgreich überwunden werden kann.

Folgt man diesen Überlegungen, scheint es wichtig, bei der medialen Darstellung von Einzelfällen vor allem auf Erfolgsgeschichten zu achten und positive Vorbilder zu präsentieren. Auf der Ebene der Selbstwahrnehmung kann man so motivierende Aufwärtsvergleiche auslösen, die Erkrankten Hoffnung machen und sie motivieren, gegen die Krankheit anzugehen. Gleichzeitig wird ein positives Bild von Personen mit Essstörungen und ihrem Umgang mit der Krankheit gezeichnet, das sowohl direkte als auch indirekte Wirkung auf die Wahrnehmung des Krankheitsbildes in der Gesellschaft haben kann und Betroffene eher motiviert, offen mit ihrer Problematik umzugehen.

Was auf jeden Fall vermieden werden sollte, ist die Präsentation potenzieller Trigger, um Nachahmung zu verhindern – also über spezielle Magertrends in sozialen Netzwerken zu berichten oder Beispiele für gefährliche Maßnahmen zu nennen, die in Pro-Ana-Foren ausgetauscht werden. Die Problematik solcher Darstellungen wurde bereits verstärkt im Kontext von Depressionen und Suizid thematisiert (und entsprechend auch in journalistischen Ethikrichtlinien verankert), kommt aber bei anderen Formen von psychischen Erkrankungen selten zur Sprache.

## Fazit und Diskussion

Medien spielen im Rahmen einer Essstörung eine wichtige Rolle – aus Sicht von Betroffenen, der Therapeut*innen und nicht zuletzt wahrscheinlich auch in der Wahrnehmung der Öffentlichkeit. Sie sind potenzielle Mitauslöser für essgestörtes Verhalten, was bereits viele Forschungsergebnisse zeigen. Aber vor allem haben sie auch Einfluss in der Verlaufs- und Bewältigungsphase der Erkrankung, was allerdings in der Forschung bisher nicht stark genug in den Fokus gestellt wurde. Wie unter anderem die Befunde von Baumann [3] zeigen, werden Medien, neben einer eher passiven Konfrontation, vor allem während des Krankheitsverlaufs auch gezielt genutzt, und zwar sowohl, um die Symptomatik aufrechtzuerhalten (destruktives Medienhandeln), als auch, um die Krankheit zu bewältigen (konstruktives Medienhandeln). Beide Strategien müssen in Bezug auf Ursachen und Folgen noch intensiver erforscht werden, um Betroffene im Krankheitsverlauf zu unterstützen.

Gerade destruktive mediale Trends wie Pro-Ana, aber auch weniger drastische Formen wie ein stark auf Schönheit bzw. Schlanksein ausgerichteter Instagram-Feed oder Casting- und Datingformate im Fernsehen können in Bezug auf ihr Gefährdungspotenzial beschrieben und im Rahmen der Therapie bzw. auch im Kontext von Präventions- bzw. Interventionsprogrammen thematisiert werden. Der Fokus sollte allerdings nicht allein auf der Frage liegen, wie Betroffene mit potenziell problematischen Inhalten umgehen bzw. wie sie diese vermeiden können, sondern welche medialen Inhalte auch gesundheitsfördernd wirken können. Insofern sollte hier sowohl in der Forschung als auch im gesellschaftlichen Diskurs ein Umdenken erfolgen: Neben den problematischen Seiten der Medien sollte auch deren potenzielle positive Rolle betrachtet werden. Die Forschung sollte beleuchten, wie konstruktives Medienhandeln im Rahmen einer Essstörung aussehen und den Erkrankten nahegebracht werden kann. In der therapeutischen Praxis wird dies bereits verstärkt thematisiert^5^, Grundlagenforschung dazu fehlt aber noch weitestgehend.

Darüber hinaus scheint es aus einer kommunikationswissenschaftlichen Perspektive wichtig, sich auch mit der Darstellung von Essstörungen in den Medien stärker zu befassen, und zwar sowohl in informierenden als auch in unterhaltenden Formaten. Zum einen kann dies Aufschluss darüber geben, welches (Zerr‑)Bild von der Krankheit in unserer Gesellschaft herrscht. Zum anderen sind erkrankte Personen auch Rezipient*innen solcher Inhalte und wie bisherige Befunde nahelegen, verfolgt gerade diese Gruppe solche Berichte bzw. Darstellungen besonders intensiv.

Wenige Erkenntnisse liegen zur Rolle von Onlineangeboten und sozialen Netzwerken für den Verlauf einer Essstörung vor, hier besteht noch Forschungsbedarf. Wie die Befunde von Lochbihler^6^ nahelegen, können solche Angebote sowohl einen positiven als auch negativen Einfluss auf den Krankheitsverlauf nehmen. Die Möglichkeiten sind hier vor allem durch den aktiven Part der Nutzer*innen umfangreich. Gerade soziale Netzwerke können aufgrund der idealisierten Darstellungen von Schönheit eine verstärkende Rolle im Kontext einer Essstörung spielen. Hier ist die Entwicklung von Medienkompetenztrainings für Schulen bzw. von Interventionsprogrammen wichtig, um potenziell negativen Effekten entgegenzuwirken [40]. Gleichzeitig können sich Erkrankte hier austauschen bzw. durch das Teilen der Erkrankung einen aktiven Umgang damit lernen.

## Footnotes

1 Lochbihler N (2017) Welche Rolle spielen massenmediale Inhalte beim Bewältigungsprozess von Essstörungen aus Expertensicht? Unveröffentlichte Bachelorarbeit, Ludwig-Maximilians-Universität München.

2 Lochbihler N (2017) Welche Rolle spielen massenmediale Inhalte beim Bewältigungsprozess von Essstörungen aus Expertensicht? Unveröffentlichte Bachelorarbeit, Ludwig-Maximilians-Universität München.

3 Lochbihler N (2017) Welche Rolle spielen massenmediale Inhalte beim Bewältigungsprozess von Essstörungen aus Expertensicht? Unveröffentlichte Bachelorarbeit, Ludwig-Maximilians-Universität München.

4 Bei Assimilationseffekten verhält es sich genau umgekehrt, sodass zum Beispiel der Vergleich mit einer besser gestellten Person positiv bzw. motivierend erlebt wird, weil deren Erfolg für einen selbst auch erreichbar erscheint.

5 Lochbihler N (2017) Welche Rolle spielen massenmediale Inhalte beim Bewältigungsprozess von Essstörungen aus Expertensicht? Unveröffentlichte Bachelorarbeit, Ludwig-Maximilians-Universität München.

6 Lochbihler N (2017) Welche Rolle spielen massenmediale Inhalte beim Bewältigungsprozess von Essstörungen aus Expertensicht? Unveröffentlichte Bachelorarbeit, Ludwig-Maximilians-Universität München.
